# An Epidemiological Study of the Risk Factors of Bicycle-Related Falls Among Japanese Older Adults

**DOI:** 10.2188/jea.JE20180162

**Published:** 2019-12-05

**Authors:** Ryota Sakurai, Hisashi Kawai, Hiroyuki Suzuki, Susumu Ogawa, Hunkyung Kim, Yutaka Watanabe, Hirohiko Hirano, Kazushige Ihara, Shuichi Obuchi, Yoshinori Fujiwara

**Affiliations:** 1 Research Team for Social Participation and Community Health, Tokyo Metropolitan Institute of Gerontology, Tokyo, Japan; 2 Research Team for Human Care, Tokyo Metropolitan Institute of Gerontology, Tokyo, Japan; 3 Research Team for Promoting Independence of the Elderly, Tokyo Metropolitan Institute of Gerontology, Tokyo, Japan; 4 Department of Social Medicine, Hirosaki University Graduate School of Medicine, Aomori, Japan

**Keywords:** older adults, cyclist, bicycling, falls

## Abstract

**Background:**

Considering the rate of growth of the older population in several countries, accidental falls in older cyclists are expected to increase. However, the prevalence and correlates of bicycle-related falls (BR-falls) are unknown. The aim of the present study was to explore the characteristics of BR-falls, focusing on the risk factors.

**Methods:**

Seven-hundred and ninety-one older adults participated in a comprehensive baseline assessment that included questions on bicycle use, BR-falls, lifestyle, and physical and cognitive evaluations. A cyclist was defined as a person who cycled at least a few times per month. The incidence of BR-falls in participants who did not report BR-falls at baseline was again ascertained 3 years later. Logistic regression analyses examined the predictors of BR-falls incidence.

**Results:**

At baseline, 395 older adults were cyclists and 45 (11.4%) of them had experienced BR-falls. Adjusted regression analysis showed that slower gait velocity, shorter one-leg standing time, and experience of falls (ie, non-BR-falls) were associated with BR-falls. Among the 214 cyclists who did not report BR-falls at baseline and who participated in both baseline and follow-up assessments, 35 (16.4%) cyclists experienced BR-falls during the 3-year follow-up. Adjusted regression analysis revealed that higher body mass index and non-BR-falls were predictors of future incidence of BR-falls, independent of physical function.

**Conclusions:**

Our results showed that experience of falls, irrespective of bicycling, is an independent correlate and risk factor of BR-falls. This suggests that experience of falls and BR-falls may share the same risk factors.

## INTRODUCTION

Bicycling is a convenient means of transportation and can help improve overall health. Considering that the population is aging in both developing and developed countries, particularly among Asian countries, the number of older cyclists will be expected to increase. A recent large-scale mail survey indicated that 63% of community-dwelling Japanese older adults in urban areas routinely ride bicycles.^1^ It has also been shown in a previous study that about 20% of older cyclists had mobility limitations, which was defined as difficulty in walking 1 km or climbing the stairs without using a handrail.^2^ These findings suggest that bicycling can encourage an active lifestyle and expand life space among older adults, including people with disability. This may result in the maintenance of physical and social functions.

Although bicycling includes several expected health benefits for older adults, being an older cyclist may predispose cycling older adults to accidents, particularly falling during bicycling, due to impaired abilities of older cyclists compared to young cyclists.^3^ Bicycling-related falls (BR-falls) have high injury risks and thus may lead to adverse health outcomes, such as severe abrasion and fractures. Although the understanding of the prevalence of BR-falls and related risk factors among older adults may provide suggestive information to promote an age-friendly city, the prevalence and risk factors are poorly known.

The purpose of this study was to investigate the prevalence of BR-falls and their risk factors among community-dwelling older adults, using longitudinal/observational data from the Tokyo Metropolitan Institute of Gerontology (TMIG) Longitudinal Study.

## METHODS

### Participants

Data were collected from an ongoing longitudinal study, named “Otassha”, aimed to examine the physical and cognitive functions for daily activities among community-dwelling older adults. Design and logistics have been described in detail elsewhere.^2^^,^^4^

On the basis of the annual registration of local residents, recruitment letters for health checkup were mailed to older adults aged 65 years and over, who were dwelling in an urban area (Itabashi Ward, Tokyo) from 2010. Participants were included in the analysis if they participated in the health checkup and answered a question regarding bicycle-related information, which was introduced in 2013 and 2016. In 2013, 2,022 older adults were recruited, and 2,497 participants were recruited in 2016. Participants who received additional assistance with their activities of daily living (ADL), had serious conditions or injuries (eg, stroke, heart disease, and injury-related falls), or did not complete all measurements, were excluded.

The study was conducted in accordance with the ethical standards of the Declaration of Helsinki (1983). Ethics approval was obtained from the Tokyo Metropolitan Institute of Gerontology and participants’ signed informed consents, obtained at enrollment prior to study assessments.

### Bicycle-related falls, confidence in bicycling, and bicycle use

Experience of BR-falls was defined as an unexpected event in which participants came to rest on the ground while bicycling in the past year. For subjective confidence in bicycling, participants responded “yes/no” to the question: “Do you have confidence in your bicycling?”. Participants were also asked about the frequency of bicycling; responses were categorized as “every day”, “a few times a week”, “a few times a month”, “a few times a year”, or “very rarely”. The participants who responded “every day” to “a few times a month” and “a few times a year” or “very rarely” were categorized as regular and irregular cyclists, respectively.^2^ Regular cyclists were then included in the analysis.

### Medical, psychological, physical, and cognitive variables

All participants were interviewed by a nurse who assessed their health-related characteristics, including demographics, comorbidities, history of hospitalization, medication, and body mass index (BMI). The frequency of going outdoors, fall history in the previous year without bicycling (ie, non-BR-falls), functional capacity (ie, instrumental ADL level), depression symptom, and fear of falling were also assessed by a trained interviewer. Functional capacity and depression symptoms were measured using TMIG Index of Competence (TMIG-IC)^5^ and Zung Self-Rating Depression Scale (SDS),^6^ respectively. Non-BR-falls were defined as any unintentional drops/falls to the ground or floor, excluding bicycle accidents, accidental contact with furniture, walls, or other environmental structures and sudden cardiovascular or central nervous system events (ie, falls with no relationship to bicycling).^7^

Physical ability was assessed using handgrip strength in the dominant hand, one-leg standing with eye opened, 5 m comfortable walking speed, and the timed up and go (TUG) test.^4^^,^^8^ Cognitive function was assessed using both the Mini-Mental State Examination (MMSE) and Montreal Cognitive Assessment, which are widely used tools for assessing global cognition.^9^

### Data analysis

Comparison between cyclists who experienced or did not experience BR-falls was made using the χ^2^ test for categorical variables and *t*-tests for continuous variables. Logistic regression analyses were then performed to examine the correlated factors of previous BR-fall on the basis of all resultant significant differences. Regression analyses were adjusted for age, sex, BMI, comorbidities, number of medications (ie, five or more or less than five medications), and frequency of riding a bicycle.

For cyclists who did not report BR-falls at baseline, differences in measurement items at baseline between participants who did not report BR-falls and those who reported new incidents of BR-falls at the follow-up assessment were compared in the same manner as the aforementioned analyses. Adjusted logistic regression analysis was also performed to elucidate the risk factors of incident of BR-falls on the basis of all resultant significant differences, similar to the cross-sectional analysis.

All statistical analyses were performed using IBM SPSS Statistics, version 20.0 (SPSS Inc., Chicago, IL, USA), and *P* values less than 0.05 were considered statistically significant.

## RESULTS

A total of 791 community-dwelling older adults participated in the baseline assessment in 2013. One hundred and three participants met the exclusion criteria, 55 participants had never ridden a bicycle, and 238 participants were irregular cyclists (ie, less than “a few times a year” or “very rarely”). In total, 395 older adults (54.4% female) with a mean age of 72.3 (standard deviation, 5.5) years were included in the cross-sectional analysis.

At the baseline, 45 participants (11.4% of regular cyclists) had experience of BR-falls. These falls were mainly due to stumbling on uneven surfaces and the curb (31.1%), losing their balance due to an operational error (31.1%), or, being surprised by or avoiding pedestrians or cars (22.2%). Compared with cyclists who did not experience BR-falls, those who experienced BR-falls showed slower gait velocity, longer TUG time, shorter one-leg standing time, higher SDS score, more fear of falling, and more experience of non-BR-falls (Table 1). There were no significant differences in subjective confidence in bicycling and frequency of riding a bicycle between the groups (with tendency of the high frequency in those who experienced BR-falls).

**Table 1.** Characteristics and differences in measurements of cyclists with and without bicycle-related falls (BR-falls) at baseline

Variables	Cyclists without BR-falls (*n* = 350)	Cyclists with BR-falls (*n* = 45)	*P*-value
Female, *n* (%)	192 (54.9)	23 (51.1)	0.635
Age, mean (SD)	72.2 (5.2)	73.2 (6.0)	0.251
BMI, mean (SD)	22.8 (2.9)	23.1 (3.2)	0.619
Grip strength, kg, mean (SD)	30.2 (8.0)	28.4 (8.1)	0.164
Gait velocity, m/s, mean (SD)	1.42 (0.21)	1.30 (0.20)	<0.001
TUG, s, mean (SD)	5.4 (0.9)	5.8 (1.0)	0.002
One-leg standing, s, mean (SD)	47.2 (20.0)	33.6 (23.8)	<0.001
TMIG-IC, mean (SD)	12.4 (1.0)	12.2 (1.1)	0.116
SDS, mean (SD)	30.0 (7.0)	32.6 (8.9)	0.023
MMSE, mean (SD)	28.5 (1.8)	28.5 (1.6)	0.801
MoCA, mean (SD)	24.4 (3.5)	23.8 (3.7)	0.303
Hypertension, yes, *n* (%)	145 (41.4)	23 (51.1)	0.216
Cerebrovascular disorder, yes, *n* (%)	21 (6.0)	1 (2.2)	0.298
Diabetes mellitus, yes, *n* (%)	40 (11.4)	7 (15.6)	0.421
Osteoporosis, yes, *n* (%)	31 (8.9)	2 (4.4)	0.314
Five plus medications, yes, *n* (%)	78 (22.3)	13 (28.9)	0.322
Fear of falling, yes, *n* (%)	100 (28.6)	24 (53.3)	0.001
Non-BR-falls, yes, *n* (%)	35 (10.0)	26 (57.8)	<0.001
Confidence in bicycling, yes, *n* (%)	322 (92.0)	41 (91.1)	0.837
Frequency of riding a bicycle, *n* (%)			0.348
Everyday	193 (55.1)	27 (60.0)	
A few times a week	106 (30.3)	15 (33.3)	
A few times a month	51 (14.6)	3 (6.7)	

BMI, body mass index; MMSE, Mini-Mental State Examination; MoCA, Montreal Cognitive Assessment; Non-BR-falls, fall history in previous year without bicycling; SD, standard deviation; SDS, Zung Self-Rating Depression Scale; TUG, timed up & go; TMIG-IC, Tokyo Metropolitan Institute of Gerontology Index of Competence.

Table 2 shows the results of the logistic regression analysis performed to examine correlated factors of previous BR-falls on the basis of all resultant significant differences. After adjusting for potential covariates, slower gait velocity, shorter one-leg standing time, and non-BR-falls were associated with BR-falls.

**Table 2.** Logistic regression analysis of factors associated with the experience of bicycle-related falls (BR-falls)

Variables	OR (95% CI)	*P*-value
Gait velocity, one m/s decrement	10.0 (1.23–85.3)	0.031
TUG, one second increment	1.08 (0.69–1.71)	0.738
One-leg standing, one second decrement	1.02 (1.00–1.04)	0.044
SDS, one increment	1.01 (0.96–1.05)	0.844
Fear of falling, yes	2.00 (0.86–4.67)	0.107
Non-BR-falls, yes	11.5 (5.26–25.0)	<0.001

CI, confidence interval; Non-BR-falls, fall history in previous year without bicycling; OR, odds ratio; SDS, Zung Self-Rating Depression Scale; TUG, timed up & go.

Model was adjusted for age, sex, BMI, comorbidities, number of medication, and frequency of riding a bicycle.

Among 395 participants included in the cross-sectional analysis, 240 participated in the follow-up assessment in 2016. Of these 240 participants, 26 were excluded because they experienced BR-falls at the baseline assessment. Among these 26 cyclists, 17 cyclists (65.4%) reported BR-falls during the 3-year follow-up. In total, 214 participants were included in the longitudinal analysis.

At the follow-up assessment, 35 participants had incident BR-falls (16.4% of cyclists who did not report BR-falls at baseline). Among cyclists who experienced new BR-falls during the 3-year follow-up, 11 (31.4%) were treated at the hospital for their injury. Comparison analyses using baseline variables showed that cyclists who experienced new BR-falls showed higher BMI, shorter one-leg standing time, lower TMIG-IC score, and more non-BR-falls (Table 3). There was no significant difference in subjective confidence between the groups. Further, the difference in frequency of riding a bicycle between the groups was also not significant; however, the percentage of frequent bicycle riders (ie, those who bicycled everyday) tended to be higher in cyclists who experienced new BR-falls.

**Table 3.** Baseline characteristics and differences of measurements of the cyclists with newly incident of bicycle-related falls (BR-falls) and those who never reported BR-falls at the follow-up assessment

Variables	Cyclists without new incident of BR-falls (*n* = 179)	Cyclists with new incident of BR-falls (*n* = 35)	*P*-value
Female, *n* (%)	104 (58.1)	25 (71.4)	0.098
Age, mean (SD)	72.0 (5.1)	72.4 (4.4)	0.256
BMI, mean (SD)	22.5 (2.7)	23.9 (4.3)	0.001
Grip strength, kg, mean (SD)	29.9 (7.5)	28.4 (6.5)	0.093
Gait velocity, m/s, mean (SD)	1.45 (0.20)	1.44 (0.26)	0.789
TUG, s, mean (SD)	5.3 (0.8)	5.3 (0.9)	0.325
One-leg standing, s, mean (SD)	50.7 (17.4)	42.7 (22.1)	0.001
TMIG-IC, mean (SD)	12.6 (0.8)	12.3 (1.3)	0.002
SDS, mean (SD)	29.0 (6.2)	30.9 (7.8)	0.122
MMSE, mean (SD)	28.7 (1.6)	28.6 (1.4)	0.701
MoCA, mean (SD)	24.9 (3.1)	24.6 (3.9)	0.105
Hypertension, yes, *n* (%)	69 (38.5)	15 (42.9)	0.384
Cerebrovascular disorder, yes, *n* (%)	14 (7.8)	1 (2.9)	0.259
Diabetes mellitus, yes, *n* (%)	16 (8.9)	4 (11.4)	0.420
Osteoporosis, yes, *n* (%)	22 (12.3)	5 (14.3)	0.462
Five plus medications, yes, *n* (%)	35 (19.6)	10 (28.6)	0.165
Fear of falling, yes, *n* (%)	40 (22.3)	12 (34.3)	0.101
Non-BR-falls, yes, *n* (%)	11 (6.1)	6 (17.1)	0.040
Confidence in bicycling, yes, *n* (%)	167 (93.3)	31 (88.6)	0.254
Frequency of riding a bicycle, *n* (%)			0.069
Everyday	97 (54.2)	24 (68.6)	
A few times a week	50 (27.9)	10 (28.5)	
A few times a month	32 (17.9)	1 (2.9)	

BMI, body mass index; MMSE, Mini-Mental State Examination; MoCA, Montreal Cognitive Assessment; Non-BR-falls, fall history in previous year without bicycling; SD, standard deviation; SDS, Zung Self-Rating Depression Scale; TUG, timed up & go; TMIG-IC, Tokyo Metropolitan Institute of Gerontology Index of Competence.

Table 4 shows the results of logistic regression analysis to examine predictive factors of future incident of BR-falls. The adjusted logistic regression analysis using all resultant significant differences showed that higher BMI and non-BR-falls were independent predictors of future incident of BR-falls.

**Table 4.** Logistic regression analysis of factors associated with a future incident of bicycle-related falls (BR-falls)

Variables	OR (95% CI)	*P*-value
BMI, one increment	1.17 (1.02–1.35)	0.035
One-leg standing, one second decrement	1.02 (1.00–1.04)	0.121
TMIG-IC, one decrement	1.49 (0.97–2.30)	0.070
Non-BR-falls, yes	5.62 (1.65–19.1)	0.006

BMI, body mass index; CI, confidence interval; Non-BR-falls, fall history in previous year without bicycling; OR, odds ratio; TMIG-IC, Tokyo Metropolitan Institute of Gerontology Index of Competence.

Model was adjusted for age, sex, comorbidities, number of medication, and frequency of riding a bicycle.

## DISCUSSION

The present study showed that 11.4% of older cyclists experienced BR-falls, while 16.4% of older cyclists who had not experienced BR-falls at the baseline newly fell while bicycling over the 3-year follow-up. Among those with incident BR-falls during the follow-up, one in three cyclists had a serious injury that required treatment at the hospital. These results suggest that bicycling is an accessible mode of transportation that comes with a high risk of failure, and it is a source of serious injury among older adults. Further, our results showed that experience of falls not related to bicycling (ie, non-BR-falls) is a risk factor of BR-falls. In other words, we found that even older adults who were likely to fall rode a bicycle regularly.

In the cross-sectional analysis, slower gait velocity and shorter one-leg standing time, as well as non-BR-falls were associated with experience of BR-falls. This is a reasonable result since the ability to ride a bicycle requires sufficient physical abilities (eg, muscle strength, balance, and flexibility) to ride stably with appropriate body and limb control. Older cyclists with lower physical abilities may thus experience previous BR-falls.

The longitudinal association between non-BR-falls and BR-falls was independent of decreased physical functioning, such as slow gait and poor balance. This robust association suggests that these falls may share the same risk factors, other than the variables measured in this study. For example, a recent study showed that compared with young adults, the increased co-contraction of the upper limb among older cyclists is higher during perturbed cycling,^10^ suggesting that this motor-control skill may be associated with increased BR-falls. Also, increased fall risk is associated with elevated co-contraction in the ankle during static balance challenges in older adults.^11^ These deficits in limb control may contribute to incidents of both BR-falls and non-BR-falls.

Our result also showed that increased BMI was an independent predictor of future incidents of BR-falls. This is somewhat reasonable because the overweight cyclist is thought to require sufficient physical abilities to ride a bicycle compared with non-overweight cyclists, due to the increased load. Furthermore, impaired gait and postural control and increased risk of falling have been reported in obese individuals.^12^ Although we did not observe significant physical impairments, older adults with higher BMI could lack physical abilities required for bicycling.

The practical implication of the present study is that, although bicycling can provide health benefits by expanding life space and by fostering the physical/social functions of older adult, caution should be taken when older adults who have experienced falls, independent of bicycling, attempt to use a bicycle. Since 65.4% of older cyclists reported recurrent BR-falling, cyclists who have experienced BR-falls should also be careful while bicycling. Although our study area, located in Itabashi Ward, Tokyo, has a relatively narrow footpath, bicycle riders generally use the footpath due to the heavy traffic congestion along the roads. Also, considering the result that the percentage of frequent bicycle riders tended to be higher in cyclists who experienced new BR-falls, bicycle lanes and other human-powered vehicles designed for fall prevention, such as tricycles, are important for the safety and comfort of such older cyclists.

To our knowledge, this is the first study to provide information on the characteristics of BR-falls (ie, the prevalence, incidence, and its risk factors) among older adults, which can provide basis for the prevention of accidents among older cyclists. However, differences in the environmental factors and the traffic regulations between Japan and other countries (eg, a bicycle can pass on both sides of a footpath in Japan), as well as the high-functionality of the participants in the present study may limit the generalizability of our results. Further multi-country research is needed to confirm the results of the present study in diverse populations.

In conclusion, we found that about one in five older cyclists newly fell while bicycling over the 3-year follow-up. Our results also showed that experience of falls unrelated bicycling was a significant risk factor of BR-falls, which is independent of decreased physical functioning. This implies that motor and cognitive factors not measured in this study might have influenced both non-BR-falls and BR-falls.
